# Kaposi’s Sarcoma-Associated Herpesvirus, but Not Epstein-Barr Virus, Co-infection Associates With Coronavirus Disease 2019 Severity and Outcome in South African Patients

**DOI:** 10.3389/fmicb.2021.795555

**Published:** 2022-01-06

**Authors:** Melissa J. Blumenthal, Humaira Lambarey, Abeen Chetram, Catherine Riou, Robert J. Wilkinson, Georgia Schäfer

**Affiliations:** ^1^International Centre for Genetic Engineering and Biotechnology (ICGEB), Cape Town, South Africa; ^2^Institute of Infectious Disease and Molecular Medicine (IDM), Faculty of Health Sciences, University of Cape Town, Cape Town, South Africa; ^3^Department of Integrative Biomedical Sciences, Faculty of Health Sciences, University of Cape Town, Cape Town, South Africa; ^4^Wellcome Centre for Infectious Disease Research in Africa, University of Cape Town, Cape Town, South Africa; ^5^Division of Medical Virology, Department of Pathology, University of Cape Town, Cape Town, South Africa; ^6^Department of Infectious Diseases, Imperial College London, London, United Kingdom; ^7^The Francis Crick Institute, London, United Kingdom

**Keywords:** KSHV, EBV, HIV, COVID-19, lytic reactivation, SARS-CoV-2, South Africa

## Abstract

In South Africa, the Coronavirus Disease 2019 (COVID-19) pandemic is occurring against the backdrop of high Human Immunodeficiency Virus (HIV), tuberculosis and non-communicable disease burdens as well as prevalent herpesviruses infections such as Epstein-Barr virus (EBV) and Kaposi’s sarcoma-associated herpesvirus (KSHV). As part of an observational study of adults admitted to Groote Schuur Hospital, Cape Town, South Africa during the period June–August 2020 and assessed for Severe Acute Respiratory Syndrome Coronavirus 2 (SARS-CoV-2) infection, we measured KSHV serology and KSHV and EBV viral load (VL) in peripheral blood in relation to COVID-19 severity and outcome. A total of 104 patients with PCR-confirmed SARS-CoV-2 infection were included in this study. 61% were men and 39% women with a median age of 53 years (range 21–86). 29.8% (95% CI: 21.7–39.1%) of the cohort was HIV positive and 41.1% (95% CI: 31.6–51.1%) were KSHV seropositive. EBV VL was detectable in 84.4% (95% CI: 76.1–84.4%) of the cohort while KSHV DNA was detected in 20.6% (95% CI: 13.6–29.2%), with dual EBV/KSHV infection in 17.7% (95% CI: 11.1–26.2%). On enrollment, 48 [46.2% (95% CI: 36.8–55.7%)] COVID-19 patients were classified as severe on the WHO ordinal scale reflecting oxygen therapy and supportive care requirements and 30 of these patients [28.8% (95% CI: 20.8–38.0%)] later died. In COVID-19 patients, detectable KSHV VL was associated with death after adjusting for age, sex, HIV status and detectable EBV VL [*p* = 0.036, adjusted *OR* = 3.17 (95% CI: 1.08–9.32)]. Furthermore, in HIV negative COVID-19 patients, there was a trend indicating that KSHV VL may be related to COVID-19 disease severity [*p* = 0.054, unstandardized co-efficient 0.86 (95% CI: –0.015–1.74)] in addition to death [*p* = 0.008, adjusted *OR* = 7.34 (95% CI: 1.69–31.49)]. While the design of our study cannot distinguish if disease synergy exists between COVID-19 and KSHV nor if either viral infection is indeed fueling the other, these data point to a potential contribution of KSHV infection to COVID-19 outcome, or SARS-CoV-2 infection to KSHV reactivation, particularly in the South African context of high disease burden, that warrants further investigation.

## Introduction

Coronavirus disease 2019 (COVID-19), the disease resulting from infection with the Severe Acute Respiratory Syndrome Coronavirus 2 (SARS-CoV-2), emerged in December 2019 and rapidly reached global proportions, officially declared a “pandemic” in March 2020 (Hui et al., 2020; World Health Organization, 2020). Since, the devastating COVID-19 pandemic has caused more than 212 million infections and 4.43 million deaths worldwide (Ritchie et al., 2020). Importantly, in countries such as South Africa with high numbers of people living with Human Immunodeficiency Virus (HIV-1), significant burdens of *Mycobacterium tuberculosis* (Mtb) and non-communicable diseases, the intersecting COVID-19 pandemic poses a significant public health crisis. Furthermore, latent oncogenic herpesvirus infections such as Epstein-Barr virus (EBV) and Kaposi’s sarcoma-associated herpesvirus (KSHV) are highly prevalent in South Africa (Sitas et al., 1999; Wilkinson et al., 1999; Schaftenaar et al., 2014; Blumenthal et al., 2018, 2019).

Mounting evidence points to potential interplay between SARS-CoV-2 infection and reactivation of opportunistic herpesvirus infections. This has been demonstrated for EBV, suggesting that reactivation of underlying EBV infection may contribute to COVID-19 symptoms, severity and time to recovery (Chen T. et al., 2021; Gold et al., 2021; Paolucci et al., 2021; Saade et al., 2021). In addition, secondary reactivation of herpes simplex virus (HSV) and Cytomegalovirus (CMV) has been reported in patients admitted to ICU with severe COVID-19 (Saade et al., 2021). Furthermore, SARS-CoV-2 encoded proteins have been shown to induce KSHV lytic reactivation *in vitro* (Chen J. et al., 2021).

While EBV infection is considered ubiquitous (Rochford, 2009), the prevalence of KSHV varies geographically and is particularly high in sub-Saharan Africa (seroprevalence 30–50%) and the Mediterranean region (20–30%) (de Sanjose et al., 2009; Mesri et al., 2010; Blumenthal et al., 2019). EBV and KSHV, both gamma-herpesviruses, have oncogenic potential, particularly in immunosuppressed patients (Schäfer et al., 2015). EBV is causally associated with Burkitt’s lymphoma, Hodgkin’s lymphoma, T and NK cell lymphomas, immunosuppression-related lymphoma, nasopharyngeal carcinoma and stomach carcinoma (Thompson and Kurzrock, 2004; Schäfer et al., 2015). KSHV is the causative agent of Kaposi’s Sarcoma, multicentric Castleman disease and primary effusion lymphoma (Mesri et al., 2010). Additionally, a lytic KSHV syndrome referred to as KSHV-related inflammatory cytokine syndrome (KICS) has been recently described (Uldrick et al., 2010; Polizzotto et al., 2012) which presents with generalized inflammatory symptoms and cytokine storm clinically akin to that of severe COVID-19 (Hu et al., 2021).

As an airborne virus, curbing SARS-CoV-2 transmission has posed a major challenge globally despite stringent travel restrictions and national and regional lock downs. This has been further exacerbated by the emergence of new highly transmissible variants of concern (van Oosterhout et al., 2021). Despite widespread vaccination programs being implemented globally, COVID-19 is likely to persist for years to come, be it due to emerging variants, transmission among unvaccinated subpopulations or waning vaccine efficacy (Phillips, 2021). The long-term effects of SARS-CoV-2 infection on virus-associated cancers, particularly in regions with high underlying EBV, KSHV, and HIV prevalence, are currently unknown and may present a public health challenge that outlasts the pandemic.

We herein present observational data on the association of KSHV and EBV co-infection on COVID-19 severity and outcome in a cross-sectional study of hospitalized COVID-19 patients recruited during the first COVID-19 wave in South Africa.

## Materials and Methods

### Study Cohort

A cohort of 104 hospitalized adult patients with confirmed acute COVID-19 (by RT-PCR) were recruited to the HIATUS (SARS-CoV-2, HIV-1, and *M. tuberculosis*) study (Riou et al., 2021) from Groote Schuur Hospital in Cape Town, South Africa between June and August 2020, during South Africa’s first wave of COVID-19 disease. The clinical characteristics of patients included in this study are presented in Table 1.

**TABLE 1:** Baseline characteristics of COVID-19 patients (*n* = 104).

Demographic information	N (%) or Median (range)	
Male sex	63 (60.6%)	
Age (years)	53.0 (21.2–85.7)	
**Virological information**	**N (%) or Median (range)**	
SARS-CoV-2 PCR positive	104 (100%)	
SARS-CoV-2 antibody positive	72 (69.2%)	
COI^*a*^	7.07 (0.06–83.03)	
HIV positive	31 (29.8%)	
Receiving ART	23 (74.2%)	
HIV VL (copies/ml)	20 (20–523,463)	
CD4 (cells/μl)	135 (3–1,367)	
KSHV seropositive	39 (41.1%)	
KSHV VL detectable in blood sample	21 (20.6%)	
KSHV VL (copies/10^6^ cells)	1.0 (1.0–38784.0)	
EBV VL detectable in blood sample	81 (84.4%)	
EBV VL (copies/10^6^ cells)	1152.0 (1.0–1.44 × 10^6^)	
KSHV and EBV infection	17 (17.7%)	
**Comorbidities**	**(N,%)**	
Tuberculosis	15 (14.4%)	
Diabetes	41 (39.4%)	
Hypertension	50 (48.1%)	
Obesity	32 (30.8%)	

**Laboratory abnormalities**	**Abnormal^*b*^ [N (%)]**	**Median (range)**

C-reactive protein (mg/l)	97 (94.2%)	170 (6–467)
D-dimer (μg/ml)	89 (89.9%)	0.6750 (0.2–5.26)
LDH (U/l)	97 (97%)	396.5 (148.0–894.0)
Ferritin (ng/ml)	93 (91.2%)	1571.0 (65.0–4217.0)
Sodium (mmol/l)	42 (46.2%)	136.0 (119.0–148.0)
Potassium (mmol/l)	12 (13.3%)	4.35 (3.2–6.6)
Hemoglobin (g/dl)	46 (45.1%)	12.5 (5.8–17.2)
White cell count (×10^9^/l)	51 (49.0%)	10.9 (2.64–33.7)
Neutrophils (×10^9^/l)	46 (57.5%)	7.4 (2.1–26.9)
Lymphocytes (×10^9^/l)	43 (53.8%)	1.2 (0.40–3.1)
Eosinophils (×10^9^/l)	0 (0%)	0.0 (0.0–0.45)
Monocytes (×10^9^/l)	Low: 21 (26.3%) High: 10 (12.5%)	0.5 (0.0–1.5)
Creatinine (μmol/l)	Low: 35 (34.3%) High: 22 (21.4%)	78.5 (35.0–374.0)
Platelets (×10^9^/l)	Low: 18 (17.6%) High: 19 (18.6%)	272.0 (32.0–679.0)
**Severity and outcome**	**N (%) or Median (range)**	
WHO score on enrollment: severe (≥ 5)	48 (46.2%)	
PC1 severity^*c*^	0.09 (–3.01 to 4.07)	
Outcome: died	30 (28.8%)	

*Data are presented as number and percentage of total or median and range, as appropriate. Missing data are excluded per characteristic.*

*^a^SARS-CoV-2 serology was performed using the Roche Elecsys^®^ assay, measuring SARS-CoV-2 nucleocapsid-specific antibodies.*

*^b^Abnormal refers to elevated C-reactive protein (> 10 mg/l); elevated D-dimer (> 0.5 μg/ml); elevated LDH (> 250U/l); elevated ferritin (males > 300 ng/ml; females > 200 ng/ml); low sodium (< 135 mmol/l); elevated potassium (> 5 mmol/l); low hemoglobin (females < 12 g/dl; males < 13 g/dl); low white cell count (< 3.9 × 10^9^/l); elevated neutrophils (males > 6.98 × 10^9^/l; females > 8.3 × 10^9^/l); low lymphocytes (< 1.4 × 10^9^/l); elevated eosinophils (females > 0.4 × 10^9^/l; males > 0.95 × 10^9^/l); low (females < 0.2 × 10^9^/l; males < 0.3 × 10^9^/l) or elevated (> 0.8 × 10^9^/l) monocytes; low (females < 49μmol/l; males < 64μmol/l) or elevated (females > 90μmol/l; males > 104μmol/l) creatinine; and low (< 186 × 10^9^/l) or elevated (females > 454 × 10^9^/l; males > 388 × 10^9^/l) platelet count.*

**FIGURE 1:**
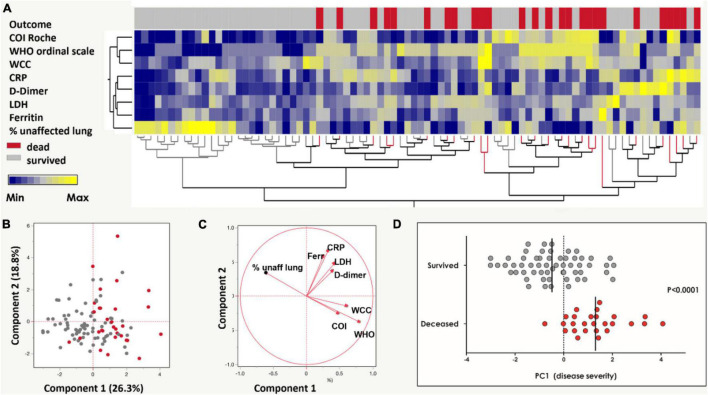
Grading of COVID-19 disease severity among the SARS-CoV-2 infected cohort (*n* = 104). **(A)** A two-way hierarchical cluster analysis using the WHO ordinal scale, anti-SARS-CoV-2 antibody cut-off index (COI, Roche Elecsys^®^), white cell count (WCC), C-reactive protein (CRP), D-dimer, Ferritin, lactate dehydrogenase (LDH) and radiographic evidence of disease extent (expressed as% of unaffected lung) was used to grade COVID-19 disease by outcome (patients survived in gray and deceased in red). Data are depicted as a heatmap colored from minimum to maximum values detected for each parameter. **(B)** Principal component analysis (PCA) based on the eight clinical parameters (as in **A**) was used to explain the variance of the data distribution in the cohort. Each dot represents a participant; 20 participants with missing data were excluded. The two axes represent principal components 1 (PC1) and 2 (PC2). Their contribution to the total data variance is shown as a percentage. **(C)** Loading plot showing each parameter’s influence on PC1 and PC2. **(D)** Comparison of PC1 scores between patients with COVID-19 who survived and died (reproduced from Riou et al., 2021). Bars represent medians and *P*-value is by the non-parametric Mann-Whitney test.

*^c^PC1 severity score refers to the calculated grading of COVID-19 disease (see Figure 1).*

*ART, antiretroviral treatment; COI, Cut-off index of Roche Elecsys^®^ assay; CRP, C-Reactive protein; LDH, lactate dehydrogenase; VL, viral load.*

The study was conducted according to the declaration of Helsinki, conformed to South African Good Clinical Practice guidelines, and was approved by the University of Cape Town’s Health Sciences Research Ethical Committee (HREC 207/2020).

### Clinical Data

Clinical and demographic details including patient co-morbidities were collected at enrollment. Absolute CD4 count (for HIV-1-infected patients) and white cell counts (WCC) were obtained from patients’ medical files. Full blood count and differential cell count, C-reactive protein (CRP), Ferritin, D-dimer, Lactate dehydrogenase (LDH), blood electrolytes, tuberculosis Gene Xpert nucleic amplification testing, and HIV-1 ELISA and viral load (VL) tests were performed by the National Health Laboratory Services, as well as SARS-CoV-2 diagnostic RT-PCR and nucleocapsid-specific IgG (see “SARS-CoV-2 detection”). Posteroanterior chest radiographs were assessed for the total percentage of the lung fields unaffected by any visible pathology.

Clinical, demographic and experimental data were recorded and stored on an electronic REDCap database (Harris et al., 2009), hosted by the University of Cape Town.

### Severe Acute Respiratory Syndrome Coronavirus 2 Detection

Diagnostic RT-PCR (Seegene, Roche or Gene Xpert) for SARS-CoV-2 was performed using nasopharyngeal or oropharyngeal aspirates sampled at the time of enrollment. SARS-CoV-2 specific antibodies were assayed by the Elecsys^®^ Anti-SARS-CoV-2 immunoassay (Roche Diagnostics). The assay was interpreted according to the manufacturer’s instructions (Roche: V 1.0 2020-05).

### Quantifying Coronavirus Disease 2019 Severity

On enrollment, patients’ COVID-19 severity based on clinical status was assessed according to the WHO ordinal scale (WHO, 2020). Briefly, patients were classified as: WHO 2: Ambulatory with limitation of activities; WHO 3: Hospitalized without requiring oxygen therapy; WHO 4: Hospitalized with oxygen required by mask or nasal prongs; WHO 5: Hospitalized and requiring non-invasive ventilation or high-flow oxygen; WHO 6: Hospitalized and receiving invasive mechanical ventilation; or WHO 7: Hospitalized and receiving invasive mechanical ventilation and additional organ support.

Additionally, a COVID-19 severity score (“PC1 severity”) was calculated using clinical indicators associated with COVID-19 severity, as previously described (Riou et al., 2021). Briefly, eight clinical parameters, namely WHO ordinal scale scoring, Roche Elecsys^®^ anti-SARS-CoV-2 antibody cut-off index (COI), WCC, CRP, D-dimer, Ferritin, LDH and radiographic evidence of disease, were graded in a non-supervised two-way hierarchical clustering analysis (HCA, ward method) segregated by outcome (died or survived). Principal component analysis was performed using the eight clinical parameters described to produce the “PC1 severity score.”

### Kaposi’s Sarcoma-Associated Herpesvirus and Epstein-Barr Virus Virological Assays

KSHV serology and KSHV and EBV VL assays were performed for all patients. Cryopreserved plasma was tested by enzyme-linked immunosorbent assay (ELISA) for antibodies against a lytic structural glycoprotein (K8.1) and latency-associated nuclear antigen (open reading frame [ORF] 73), following established specifications (Mbisa et al., 2010), and patients were considered KSHV seropositive if antibodies to either antigen were detected.

To perform VL assays, DNA was extracted from whole blood with plasma removed using the QIAamp DNA Blood Midi kit (Qiagen). KSHV and EBV DNA were quantified by real-time qPCR targeting the KSHV K6 gene (De Sanjosé et al., 2002) and EBV polymerase gene (Labo et al., 2019), respectively. Each reaction was performed in triplicate with 250 ng input DNA, 100 pmole forward and reverse primers, 5 pmole FAM/TAMRA labeled probe and 2X Universal Master Mix (Applied Biosystems). DNA was quantified against standard curves constructed by serial dilution of a K6 or EBV-pol plasmid. Cycling conditions on a LightCycler 480II System (Roche) were as follows: 2 min at 50°C; 8 min at 95°C; and 45 cycles of 15 s at 95°C and 1 min at 60°C for the KSHV assay and 2 min at 50°C; 10 min at 95°C; and 45 cycles of 15 s at 95°C and 1 min at 57°C for the EBV assay. Cellular equivalents per sample were determined using a quantitative assay for human endogenous retrovirus 3 (Yuan et al., 2001) and reported as viral DNA copies per million cells. Samples that failed to amplify in one or two replicates, or with detectable viral DNA in each replicate lower than the limit of detection for each assay (3 copies/reaction for KSHV and < 10 copies for EBV) were classified as qualitatively positive and arbitrarily assigned the value of 1 and 3 copies, respectively, as previously reported (Labo et al., 2019).

### Statistical Analysis

Statistical analysis was performed in SPSS version 25 (IBM Corp., 2017). Graphical representations were performed in Prism (v5; GraphPad Software Inc., San Diego, CA, United States) and JMP (v15.0.0; SAS Institute, Cary, NC, United States). Univariate analyses consisted of non-parametric Wilcoxon rank-sum tests and Fisher exact tests, as appropriate. Multivariate analyses were performed using binomial logistic regression for the categorical dependent variable, “outcome,” in relation to the specified covariates. Linearity of the continuous variables with respect to the logit of the dependent variable was confirmed via the Box–Tidwell procedure (Box and Tidwell, 1962), and studentized residuals with values < 2.5 standard deviations were accepted. Multiple linear regression was performed to assess the association of categorical and continuous independent variables with the continuous dependent variable, “PC1 severity.” Continuous variables were transformed, where appropriate, to approximate normal distributions. *P*-values are 2-tailed and were considered significant if < 0.05. Participants with missing data were excluded pairwise in each analysis.

## Results

### Clinical Characteristics of the Study Participants

The clinical characteristics of the patients with RT-PCR proven SARS-CoV-2 infection included in this study (*n* = 104) are listed in Table 1.

Briefly, 61% of patients were men and 39% women with a median age of 53 years (range: 21–86). Serology assays indicated that 69.2% (95% CI: 59.9–77.5) were positive for SARS-CoV-2 antibodies and 41.1% (95% CI: 31.6–51.1%) were KSHV seropositive. About a third of the patients [29.8% (95% CI: 21.7–39.1%)] were HIV-1 positive, the majority of whom were on antiretroviral therapy [74.2% (95% CI: 57.1–87.0)]. The median HIV-1 VL among the HIV positive patients was 20 copies/ml with a range of 20–523,463 copies/ml and median CD4 count was 134 cells/μl (range: 3–1,367). EBV VL was detectable in 84.4% (95% CI: 76.1–84.4%) of the cohort with a median VL of 1,152 copies/10^6^ cells (range: 1.0–1.44 × 10^6^ copies/10^6^ cells); similarly, in a pre-pandemic South African cohort of HIV-positive controls, EBV was detectable in 78.3% (95% CI: 58.7–91.2) with a median VL of 6,885 copies/10^6^ cells (range: 1.0–1.65 × 10^6^ copies/10^6^ cells; *n* = 23; data not shown). KSHV DNA was detected in 20.6% (95% CI: 13.6–29.2%) with a median VL of 1.0 copies/10^6^ cells (range 1.0–38,784 copies/10^6^ cells). This percentage positive is significantly higher than that reported in our previous study (Blumenthal et al., 2019) [6.4% (95% CI: 4.7–8.4%); *p* < 0.0001]. Both EBV and KSHV DNA was detectable in 17.7% (95% CI: 11.1–26.2%) of the cohort. There was no correlation between HIV VL and EBV VL or KSHV VL (data not shown). EBV VL detection did not differ between HIV positive and HIV negative patients (80.8% in HIV positive vs. 85.7% in HIV negative, *p* = 0.541) but EBV VL was significantly higher among HIV positive patients (Supplementary Table 1). KSHV VL detection was greater, although not statistically significant, among HIV positive patients compared to HIV negative patients (27.5% in HIV positive vs. 17.8% in HIV negative, *p* = 0.287) and KSHV seroprevalence was significantly greater among HIV positive patients (63.3% in HIV positive vs. 30.7% in HIV negative, *p* = 0.004) with higher ORF73 OD values (Supplementary Table 1).

Most patients had an elevated CRP (94.2%), D-Dimer (89.9%), LDH (97%) and ferritin (91.2%) levels. Also of note, large proportions of COVID-19 patients exhibited abnormal hemoglobin (45.1%), WCC (49.0%), neutrophils (57.5%) and lymphocytes (53.8%).

On enrollment 48 [46.2% (95% CI: 36.8–55.7%)] COVID-19 patients were classified as severe on the WHO ordinal scale reflecting oxygen therapy and supportive care requirements. Hierarchical clustering analysis and subsequent principal component analysis based on eight clinical variables included in this study (WHO ordinal scale, Roche Elecsys^®^ anti-SARS-CoV-2 antibody COI, WCC, CRP, D-dimer, ferritin, LDH and radiographic evidence of disease extent (expressed as% of unaffected lung) showed distinct separation by COVID-19 disease outcome (Figures 1A,B). PC1 accounted for 26.3% and PC2 18.8% of the variance in the distribution. The range of PC1 severity scores in the cohort was –3.01 to 4.07 (Figure 1D, reproduced from Riou et al., 2021; Table 1). Thirty [28.8% (95% CI: 20.8–38.0%)] COVID-19 patients died and this group had a significantly higher PC1 score compared to patients who survived (*p* < 0.0001, Figure 1D, reproduced from Riou et al., 2021).

### Association of Kaposi’s Sarcoma-Associated Herpesvirus and Epstein-Barr Virus With Coronavirus Disease 2019 Severity and Outcome in the Entire Coronavirus Disease 2019 Cohort

**FIGURE 2:**
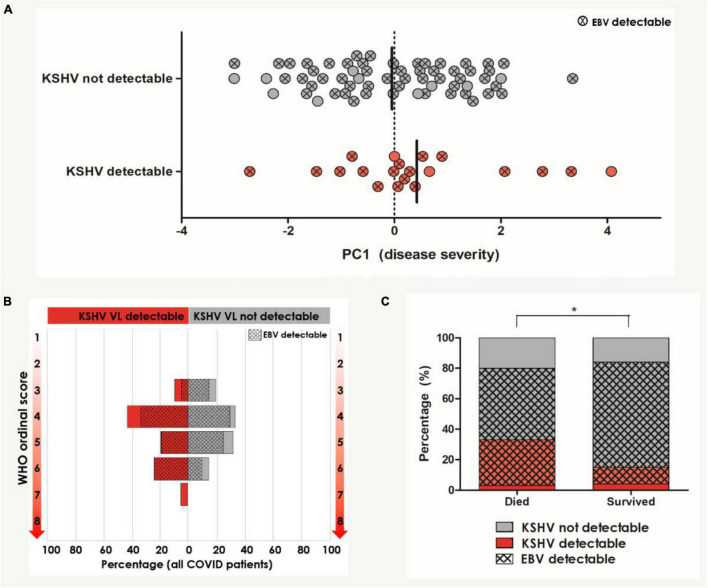
Univariate analysis of KSHV and EBV VL detection in relation to COVID-19 severity and outcome (*n* = 104). **(A)** PC1 severity score amongst patients with and without detectable KSHV VL in the blood. Circles indicated with an X represent patients who also have detectable EBV VL in the blood. Bars indicate median. **(B)** The distribution of WHO ordinal scale scores between patients with and without detectable KSHV VL. Hash pattern indicates percentages of patients with detectable EBV VL. **(C)** The distribution of patients with and without detectable KSHV VL between patients who died and survived. Hash pattern indicates patients with detectable EBV VL. *Indicates the statistically significant proportion of patients with detectable KSHV and EBV VL who died compared to those who survived (*p* = 0.039). Participants with missing data were excluded pairwise.

The association of KSHV, EBV, and the detection of both viruses with COVID-19 severity (as measured by PC1 severity score and WHO ordinal scale score) as well as outcome was first assessed in univariate analyses (Table 2 and Figure 2).

**TABLE 2:** Univariate analysis comparing virological parameters between COVID-19 patients (*n* = 104) who died and survived.

Parameter	Died (30) N (%) or Median (range)	Discharged (74) N (%) or Median (range)	*P*-value
KSHV VL detectable	10 (33.3%)	11 (15.3%)	0.059
KSHV VL (copies/10^6^ cells)	1.0 (1.0–1.0)	1.0 (1.0–38783.96)	0.314
EBV VL detectable	23 (79.3%)	58 (86.6%)	0.374
EBV VL (copies/10^6^ cells)	1018.56 (1.0–201276.1)	3.0 (1.0–1.44E6)	0.168
KSHV seropositive	6 (23.1%)	33 (47.8%)	0.036
K8.1 positive	4 (15.4%)	18 (26.1%)	0.414
ORF73 positive	5 (19.2%)	27 (39.1%)	0.089
K8.1 OD	1.51 (0.76–2.96)	1.18 (0.21–3.43)	0.391
ORF73 OD	1.31 (0.83–5.19)	2.66 (0.15–8.28)	0.227
KSHV-EBV coinfection	9 (31.0%)	8 (11.9%)	0.039

*Participants with missing data were excluded pairwise. P-values are by Fisher’s Exact test for categorical variables and Mann-Whitney U-test for categorical variables.*

*ART, antiretroviral therapy; HIV, human immunodeficiency virus; KSHV, Kaposi sarcoma-associated herpesvirus; VL, viral load; EBV, Epstein-Barr virus.*

PC1 severity score and KSHV VL did not correlate (Spearman’s rho correlation coefficient = 0.083, *p* = 0.456) but the median PC1 severity score was slightly higher in patients with detectable KSHV, although this did not reach statistical significance (*p* = 0.394, Figure 2A). EBV VL similarly did not correlate with PC1 severity (Spearman’s rho correlation coefficient = 0.065, *p* = 0.569). The distribution of WHO ordinal scale scores amongst patients with and without detectable KSHV VL and EBV VL was not significantly different (Figure 2B). While EBV was detectable in most COVID-19 patients (84.4%) with no discernable difference in detection nor VL between patients who died and those who survived (Table 2), KSHV was detected more frequently (although this trend was not significant) among the patients who died (died: 33.3% vs. survived: 15.3%, *p* = 0.059, Table 2 and Figure 2C). This, however, is not reflected in KSHV seropositivity as a greater proportion of patients who survived were indeed KSHV seropositive (*p* = 0.036). Similarly, there was an overrepresentation of detection of both KSHV and EBV among patients who died (died: 31.0% vs. survived: 11.9%, *p* = 0.039), however, this is likely due to the almost ubiquitous detection of EBV reflecting a difference in KSHV detection between groups rather than any contribution of dual detection.

Further assessment of parameters that differed between the patients who died and survived indicated that male sex, severe WHO score on enrollment, higher PC1 severity score, elevated CRP, D-dimer, LDH, Ferritin, creatinine, WCC and neutrophil count were similarly associated with death on a univariate level (Supplementary Table 2); these parameters were therefore considered in multivariate analysis. In COVID-19 patients, detectable KSHV VL was associated with death after adjusting for age, sex, HIV status, detectable EBV VL, creatinine, neutrophils and PC1 severity [Table 3A, *p* = 0.036, adjusted *OR* = 7.35 (95% CI: 1.14–47.58)]. To avoid overfitting the model, variables that were not significant in model A were removed and a stripped-down logistic regression was run confirming that detectable KSHV VL was associated with death after adjusting for sex, age and PC1 severity [Table 3B, *p* = 0.045, adjusted *OR* = 4.59 (95% CI: 1.04–20.31)].

**TABLE 3:** Logistic regression for death outcome in COVID-19 positive patients (*n* = 104).

Characteristic	Unadjusted OR	95% CI for unadjusted OR		Adjusted OR	95% CI for adjusted OR		*P*-value
		Lower	Upper		Lower	Upper	
**Model A**							
Detectable KSHV VL^a^	2.773	1.026	7.493	7.347	1.135	47.574	0.036
Detectable EBV VL^b^	0.595	0.190	1.860	0.222	0.007	6.787	0.388
Sex^c^	2.793	1.068	7.306	3.244	0.528	19.922	0.204
Age	0.969	0.934	1.006	0.996	0.920	1.079	0.930
HIV status^d^	0.490	0.177	1.354	6.507	0.595	71.129	0.125
Creatinine	0.990	0.983	0.998	0.998	0.986	1.009	0.709
Neutrophils	0.914	0.824	1.013	1.111	0.890	1.387	0.352
PC1 severity	3.546	1.961	6.410	6.757	2.024	22.727	0.002
**Model B**							
Detectable KSHV VL^a^	2.773	1.026	7.493	4.585	1.035	20.314	0.045
PC1 severity	3.546	1.961	6.410	4.219	2.033	8.772	<0.0001
Sex^c^	2.793	1.068	7.306	2.711	0.717	10.256	0.142
Age	0.969	0.934	1.006	1.004	0.948	1.063	0.892

*^a^Detectable KSHV VL is for detectable VL compared to not detectable VL.*

*^b^Detectable EBV VL is for detectable VL compared to not detectable VL.*

*^c^Sex is for male compared to female.*

*^d^HIV status is for HIV positive compared to HIV negative.*

### Association of Kaposi’s Sarcoma-Associated Herpesvirus and Epstein-Barr Virus With Coronavirus Disease 2019 Severity and Outcome in the Human Immunodeficiency Virus-1 Negative Sub-Cohort

In a further analysis, we excluded HIV-1 positive patients as PC1 severity among HIV-1 positive patients was significantly lower than in HIV negative patients (data not shown, *p* = 0.032) indicating a recruitment bias due to the presentation and hospitalization of HIV positive patients for HIV-related health issues other than COVID-19. In HIV negative COVID-19 patients, there was a trend of greater detection of KSHV VL with COVID-19 disease severity when controlling for sex and age [Table 4, *p* = 0.054, unstandardized co-efficient 0.86 (95% CI: –0.015 to 1.74)]. Additionally, detectable KSHV VL was associated with death when controlling for PC1 severity, sex and age [Table 5, *p* = 0.008, adjusted *OR* = 7.34 (95% CI: 1.69–31.49)].

**TABLE 4:** Multiple regression for PC1 severity in HIV negative COVID-19 positive patients (*n* = 73).

Characteristic	Unstandardized coefficient	Standard error	Standardized coefficient	*P*-value
Detectable KSHV VL^a^	0.864	0.439	0.253	0.054
Sex^b^	–0.041	0.395	–0.013	0.919
Age	0.035	0.017	0.269	0.042

*^a^Detectable KSHV VL is for detectable VL compared to not detectable VL.*

*^b^Sex is for male compared to female.*

**TABLE 5:** Logistic regression for death outcome in HIV negative COVID positive patients (*n* = 73).

Characteristic	Unadjusted OR	95% CI for unadjusted OR		Adjusted OR	95% CI for adjusted OR		*P*-value
		**Lower**	**Upper**		**Lower**	**Upper**	
Detectable KSHV VL^a^	4.400	1.254	15.440	23.000	2.019	261.964	0.012
PC1 severity	3.968	1.876	8.403	6.536	2.045	20.833	0.002
Sex^b^	3.167	0.937	10.701	8.385	1.170	60.083	0.034
Age	0.959	0.918	1.003	0.954	0.885	1.028	0.213

*^a^Detectable KSHV VL is for detectable VL compared to not detectable VL.*

*^b^Sex is for male compared to female.*

## Discussion

Systemic reactivation of herpesviruses has been reported in critically ill COVID-19 patients (Simonnet et al., 2021). The herein presented data support these observations and suggest an association between KSHV and COVID-19 outcome; however, it is not clear if the underlying KSHV infection is contributing to severity of COVID-19 or if SARS-CoV-2 infection is causing reactivation of KSHV. On the contrary, detection of EBV in our cohort was similar to what we have seen in a previous pre-pandemic cohort and what has been previously reported (Schaftenaar et al., 2014) and our results do not show EBV to be related to COVID-19 severity or outcome.

Previous research has demonstrated EBV lytic reactivation following SARS-CoV-2 infection (Paolucci et al., 2021). Moreover, EBV lytic reactivation was found to enhance SARS-CoV2 infection (Chen T. et al., 2021; Verma et al., 2021). While this was not evident in our cohort, possibly due to almost ubiquitous EBV detection in the South African population even before the COVID-19 pandemic, it is tempting to speculate that similar mechanisms play a role for the related herpesvirus, KSHV, in our cohort, causing some disease synergy. Indeed, we found a higher than usual detection of lytic KSHV compared to previous pre-pandemic HIV-1-infected patient cohorts from the same geographic area (Blumenthal et al., 2019), and *in vitro* studies have also suggested that SARS-CoV-2 and drugs used in COVID-19 treatment, namely Azithromycin and Nafamostat mesylate, can induce KSHV lytic reactivation (Chen J. et al., 2021). This suggests that SARS-CoV-2 infection may cause reactivation of KSHV in latently infected individuals.

We unexpectedly noted several patients with detectable KSHV VL who were KSHV seronegative. KSHV infection is generally considered to be obtained in childhood in sub-Saharan Africa, with KSHV seroprevalence peaking before adulthood (Bourboulia et al., 1998) therefore it is unlikely these cases represent new infections. Indeed, while KSHV detection is greater in this cohort than what we have seen in pre-pandemic cohorts (Blumenthal et al., 2019), viral loads are significantly lower and it is plausible that the antibody levels in these cases fall below the detection limit of our assay.

In severely ill patients, lytic KSHV infection can culminate in generalized inflammation and an IL-6 induced cytokine storm (described as KICS) (Uldrick et al., 2010; Polizzotto et al., 2012; Blumenthal et al., 2019). Similarly, a cytokine storm has been described in severely ill COVID-19 patients as a crucial cause of death (Hu et al., 2021). Further, lytically associated multicentric Castleman disease as well as primary effusion lymphoma and KS pose major diagnostic challenges globally and particularly in low resource settings due to non-specific presentation, especially in the context of high COVID-19, TB and HIV prevalence, and technically difficult diagnostic requirements. While the low global prevalence of latent KSHV infection and potentially associated disease synergy with lytic reactivation and COVID-19 severity are unlikely to represent a major public health concern, geographic regions where KSHV is highly prevalent may be faced with a rising incidence of lytic KSHV-related syndromes.

The observation that HIV-1 positive patients in our cohort presented with a lower PC1 severity score was interesting although likely reflects a recruitment bias rather than any protective effect of HIV-1. HIV negative patients were hospitalized on clinical suspicion of COVID-19 disease whereas HIV positive patents may have been hospitalized due to HIV-1-related diseases, such as TB, and found to have a concurrent SARS-COV-2 infection. Examination of COVID-19 disease in the HIV positive population in South Africa has shown HIV-1 to be independently associated with increased risk of severe COVID-19 disease and death (Boulle et al., 2020; Davies, 2020) while HIV positive patients who were virally suppressed due to ART do not have altered SARS-CoV-2 CD4 T cell function (Riou et al., 2021). Our relatively small subset of HIV positive patients with COVID-19 disease disallows us from commenting specifically on the interplay of HIV-1, KSHV/EBV and SARS-CoV-2.

Although longitudinal studies are required to support our data, our results have potential implications for future KSHV- and EBV-related disease development following the COVID-19 pandemic, particularly in regions where prevalence of these herpesviruses and HIV-1 co-infection is high. In this context, prioritization of COVID-19 vaccination in these populations should be considered and history of COVID-19 disease, even after full recovery, should be taken into account as a potential risk factor for virus-associated cancer in the future management and screening of these patients. These data support the clinical monitoring of KSHV VL both in COVID-19 disease and future management of patients with KSHV infection.

## Data Availability Statement

The original contributions presented in the study are included in the article/Supplementary Material, further inquiries can be directed to the corresponding author/s.

## Ethics Statement

The studies involving human participants were reviewed and approved by the University of Cape Town’s Faculty of Health Sciences Research Ethical Committee (HREC 207/2020). The patients/participants provided their written informed consent to participate in this study.

## Author Contributions

MB, CR, RW, and GS designed the study. CR and RW facilitated clinical recruitment. MB, HL, and AC performed the diagnostic experiments. MB, CR, and GS performed the data analysis and interpretation. MB and GS wrote the manuscript with all authors.

## Conflict of Interest

The authors declare that the research was conducted in the absence of any commercial or financial relationships that could be construed as a potential conflict of interest.

## Publisher’s Note

All claims expressed in this article are solely those of the authors and do not necessarily represent those of their affiliated organizations, or those of the publisher, the editors and the reviewers. Any product that may be evaluated in this article, or claim that may be made by its manufacturer, is not guaranteed or endorsed by the publisher.
