# Supplementary material for: Kaposi’s Sarcoma-Associated Herpesvirus, but Not Epstein-Barr Virus, Co-infection Associates With Coronavirus Disease 2019 Severity and Outcome in South African Patients
Source: Front Microbiol. 2022 Jan 6;12:795555. doi: 10.3389/fmicb.2021.795555 (PMC8770866; doi:10.3389/fmicb.2021.795555)
Supplement: Supplementary file 1 [file Table_1.docx]

| **Supplementary table 1: Univariate analysis comparing virological parameters between HIV-1 positive and HIV-1 negative COVID-19 patients.** P values are by Fisher’s Exact test for categorical variables and Mann-Whitney U test for categorical variables. | | | |
| --- | --- | --- | --- |
| **Parameter** | **HIV-1 positive (31)**  **N (%) or Median (range)** | **HIV-1 negative (73)**  **N (%) or Median (range)** | **P value** |
| KSHV VL detectable | 8 (27.6%) | 13 (17.8%) | 0.287 |
| KSHV VL (copies/10^6^ cells) | 1.0 (1.0 – 38783.96) | 1.0 (1.0 – 1.00) | 0.161 |
| EBV VL detectable | 21 (80.8%) | 60 (85.7%) | 0.541 |
| EBV VL (copies/10^6^ cells) | 3835.27 (1.0 – 1440000) | 535.15 (1.0 – 111194.9) | 0.008 |
| KSHV seropositive | 19 (63.3%) | 20 (30.8%) | 0.004 |
| K8.1 positive | 11 (36.7%) | 11 (16.9%) | 0.041 |
| ORF73 positive | 18 (60.0%) | 14 (21.5%) | <0.001 |
| K8.1 OD | 1.95 (0.23 – 3.08) | 1.05 (0.21 – 3.43) | 0.351 |
| ORF73 OD | 3.48 (0.35 – 8.22) | 1.68 (0.15 – 8.28) | 0.038 |
| KSHV-EBV coinfection | 6 (23.1%) | 11 (15.7%) | 0.387 |
| ART: antiretroviral therapy; HIV: human immunodeficiency virus; KSHV: Kaposi sarcoma-associated herpesvirus; VL: viral load; EBV: Epstein-Barr virus | | | |
