# Supplementary material for: Kaposi’s Sarcoma-Associated Herpesvirus, but Not Epstein-Barr Virus, Co-infection Associates With Coronavirus Disease 2019 Severity and Outcome in South African Patients
Source: Front Microbiol. 2022 Jan 6;12:795555. doi: 10.3389/fmicb.2021.795555 (PMC8770866; doi:10.3389/fmicb.2021.795555)
Supplement: Supplementary file 2 [file Table_2.docx]

| **Supplementary table 2: Selected parameters in PCR positive COVID-19 patients (n=104) stratified by survival.** Two patients were lost to follow up. Only P values <0.05 (considered significant) are shown. | | | | | | | | |
| --- | --- | --- | --- | --- | --- | --- | --- | --- |
|  | | **Died (30)** | | | | **Discharged (72)** | |  |
| **Demographic and severity parameters** | | **N (%) or Median (range)** | | | | **N (%) or Median (range)** | | **P value** |
| Male sex | | 23 (76.7%) | | | | 40 (54.1%) | | 0.046 |
| Age (years) | | 55.4 (30.8 – 80.1) | | | | 51.8 (21.2 – 85.7) | | ns |
| Days in medical care | | 16 (4 – 43) | | | | 12 (2 – 58) | | ns |
| Days on high-flow oxygen | | 0 (0 – 16) | | | | 0 (0 – 10) | | ns |
| Days on ventilator | | 6 (0 – 33) | | | | 0 (0 – 36) | | <0.0001 |
| WHO on enrolment: | |  | | | |  | |  |
| Mild/moderate (<5) | | 5 (16.7%) | | | | 51 (68.9%) | | <0.0001 |
| Severe (≥5) | | 25 (83.3%) | | | | 23 (31.1%) | |  |
| PC1 severity score | | 1.289 (-0.775 – 4.075) | | | | -0.560 (-3.009 – 2.057) | | <0.0001 |
| Co-morbidities: | |  | | | |  | |  |
| HIV-1 | | 6 (20,0%) | | | | 25 (33.8%) | | ns |
| TB | | 6 (20,0%) | | | | 9 (12.2%) | | ns |
| Cardiovascular disease | | 2 (6.7%) | | | | 5 (6.8%) | | ns |
| Diabetes | | 14 (46.7%) | | | | 27 (36.5%) | | ns |
| Hypertension | | 17 (56.7%) | | | | 33 (44.6%) | | ns |
| Obesity | | 10 (33.3%) | | | | 22 (29.7%) | | ns |
| Respiratory disease | | 0 (0.0%) | | | | 7 (9.5%) | | ns |
| Chronic kidney disease | | 3 (10.0%) | | | | 1 (1.4%) | | ns |
| Malignancy | | 1 (3.3%) | | | | 2 (2.7%) | | ns |
| Organ transplant recipient | | 0 (0.0%) | | | | 1 (1.4%) | | ns |
| Number of comorbidities^2^: | |  | | | |  | | ns |
| 0 | | 2 (6.7%) | | | | 9 (12.2%) | |  |
| 1 | | 10 (33.3%) | | | | 24 (32.4%) | |  |
| 2 | | 9 (30.0%) | | | | 20 (27.0%) | |  |
| 3 | | 6 (20.0%) | | | | 15 (20.3%) | |  |
| 4 | | 2 (6.7%) | | | | 6 (8.1%) | |  |
|  | |  | | | |  | |  |
|  | **Died (30)** | | | | **Discharged (72)** | | |  |
| **Selected laboratory abnormalities** | **Abnormal^1^**  **(N, %)** | | **Median (range)** | **Abnormal^1^**  **(N, %)** | | | **Median (range)** | **P value** |
| C-reactive protein | 29 (100%) | | 134.5 (18.0 – 469.0) | 68 (91.9%) | | | 55.0 (2.0 – 239.0) | 0.005 |
| D-dimer | 28 (96.6%) | | 0.85 (0.20 – 7.02) | 61 (87.1%) | | | 0.53 (0.10 – 5.36) | 0.016 |
| LDH | 28 (100%) | | 501.5 (312.0 – 879.0) | 69 (95.8%) | | | 358.0 (148.0 – 894) | 0.001 |
| Ferritin | 28 (96.6%) | | 1847 (40 – 3617) | 65 (89.0%) | | | 855.0 (65 – 4217) | 0.001 |
| Sodium | Low: 12 (44.4%)  High: 3 (11.1%) | | 138 (125 – 148) | Low: 30 (46.9%)  High: 0 (0%) | | | 136 (124 – 144) | ns |
| Potassium | Low: 0 (0%)  High: 2 (7.4%) | | 4.45 (3.50 – 5.50) | Low: 2 (3.2%)  High: 10 (15.9%) | | | 4.20 (3.20 – 6.30) | ns |
| Creatinine | Low: 5 (16.7%)  High: 11 (36.7%) | | 96.5 (35.0 – 374.0) | Low: 30 (41.1%)  High: 11 (15.1%) | | | 74.0 (39.0 – 297.0) | 0.002 |
| Haemoglobin | 12 (40.0%) | | 12.35 (7.60 – 16.90) | 34 (47.2%) | | | 12.50 (5.80 – 17.20) | ns |
| White cells | Low: 0 (0%)  High: 19 (63.3%) | | 13.01 (5.21 – 33.69) | Low: 4 (5.4%)  High: 32 (43.2%) | | | 8.68 (2.64 – 28.83) | 0,010 |
| Lymphocytes | 15 (60.0%) | | 1.26 (0.40 – 2.87) | 28 (50.9%) | | | 1.41 (0.42 – 3.05) | ns |
| Monocytes | Low: 7 (28.0%)  High: 4 (16.0%) | | 0.52 (0.06 – 1.56) | Low: 14 (25.5%)  High: 6 (10.9%) | | | 0.53 (0.00 – 0.94) | ns |
| Eosinophils | 0 (0%) | | 0.01 (0.00 – 0.14) | 0 (0%) | | | 0.03 (0.00 – 0.45) | ns |
| Neutrophils | 19 (76.0%) | | 9.93 (4.0 – 18.0) | 27 (49.1%) | | | 7.06 (2.1 – 26.9) | 0.022 |
| Platelets | Low: 7 (23.3%)  High: 5 (16.7%) | | 300 (108 – 679) | Low: 11 (15.3%)  High 14 (19.4%) | | | 277 (114 – 657) | ns |
| ^1^Abnormal refers to elevated C-reactive protein (>10mg/l); elevated D-dimer (>0.5µg/ml); elevated LDH (>250U/l); elevated ferritin (males >300ng/ml; females >200ng/ml); low sodium (<135mmol/l); elevated potassium (>5mmol/l); low haemoglobin (females <12g/dl; males <13g/dl); low white cell count (<3.9x10^9^/l); elevated neutrophils (males >6.98x10^9^/l; females >8.3x10^9^/l); low lymphocytes (<1.4x10^9^/l); elevated eosinophils (females >0.4x10^9^/l; males >0.95x10^9^/l); low (females <0.2x10^9^/l; males <0.3x10^9^/l) or elevated (>0.8x10^9^/l) monocytes; low (females <49µmol/l; males <64µmol/l) or elevated (females >90µmol/l; males >104µmol/l) creatinine; and low (<186x10^9^/l) or elevated (females >454x10^9^/l; males >388x10^9^/l) platelet count.  ^2^ Comorbidities recorded to calculate number of comorbidities include HIV-1, TB, cardiovascular disease, diabetes, hypertension, obesity, respiratory disease, chronic kidney disease, malignancy, immunosuppressive treatment and organ transplant. | | | | | | | | |
