# Supplementary material for: Kaposi’s Sarcoma-Associated Herpesvirus, but Not Epstein-Barr Virus, Co-infection Associates With Coronavirus Disease 2019 Severity and Outcome in South African Patients
Source: Front Microbiol. 2022 Jan 6;12:795555. doi: 10.3389/fmicb.2021.795555 (PMC8770866; doi:10.3389/fmicb.2021.795555)
Supplement: Supplementary file 3 [file Table_3.docx]

## Complete list of HIATUS consortium members (alphabetical order):

1. Fatimah Abrahams, University of Cape Town

2. Saalikha Aziz, University of Cape Town

3. Nonzwakazi Bangani, University of Cape Town

4. John Black, Livingstone Hospital, Port Elizabeth

5. Melissa Blumenthal, University of Cape Town

6. Marise Bremer, University of Cape Town

7. Wendy Burgers, University of Cape Town

8. Maddalena Cerrone, University of Cape Town, Imperial College London and Francis Crick Institute

9. Zandile Ciko, University of Cape Town

10. Anna K Coussens, University of Cape Town and Walter and Eliza Hall Institute of Medical Research, and University of Melbourne

11. Remy Daroowala, Imperial College London and University of Cape Town

12. Angharad G Davis, Francis Crick Institute, University of Cape Town and University College London

13. Jantina de Vries, University of Cape Town

14. Elsa du Bruyn, University of Cape Town

15. Hanif G Esmail, University College London and University of Cape Town

16. Rene T Goliath, University of Cape Town

17. Siamon Gordon, University of Oxford

18. Yolande XR Harley, University of Cape Town

19. Amanda Jackson, University of Cape Town

20. Rachel P-J Lai, Imperial College London and Francis Crick Institute, London

21. Francisco Lakay, University of Cape Town

22. Fernando-Oneissi Martinez-Estrada, University of Surrey

23. Graeme Meintjes, University of Cape Town

24. Marc S Mendelson, University of Cape Town

25. Ntobeko Ntusi, University of Cape Town

26. Tari Papavarnavas, University of Cape Town

27. Alize Proust, Francis Crick Institute, London

28. Catherine Riou, University of Cape Town

29. Sheena Ruzive, University of Cape Town

30. Qonita Said-Hartley, University of Cape Town

31. Georgia Schäfer, International Centre for Genetic Engineering and Biotechnology, Cape Town

32. Keboile Serole, University of Cape Town

33. Cari Stek, Imperial College London and University of Cape Town

34. Nicki Tiffin, University of Cape Town

35. Sean Wasserman, University of Cape Town

36. Claire Whitaker, University of Cape Town

37. Katalin A Wilkinson, Francis Crick Institute and University of Cape Town

38. Robert J Wilkinson, University of Cape Town, Imperial College London and Francis Crick Institute

39. Kennedy Zvinairo, University of Cape Town
